# Rate and risk factors of inadequate endometrial tissues after endometrial sampling among Bhutanese women at the national referral hospital of Bhutan: a cross-sectional study

**DOI:** 10.1186/s12905-024-03047-6

**Published:** 2024-04-02

**Authors:** Namkha Dorji, Yeshey Dorjey, Sangay Tshering, Birendra Pradhan, Meera Chhetri, Damanti Bhujel

**Affiliations:** 1 Department of Obstetrics and Gynaecology, Jigme Dorji Wangchuck National Referral Hospital, Thimphu, Bhutan; 2 Department of Obstetrics and Gynaecology, Phuntsholing General Hospital, Chukha, Bhutan; 3 Department of Pathology and Laboratory Medicine, Jigme Dorji Wangchuck National Referral Hospital, Thimphu, Bhutan; 4 Colposcopy Clinic, Gyaltsuen Jetsun Pema Mother and Child Hospital, Jigme Dorji Wangchuck National Referral Hospital, Thimphu, Bhutan

**Keywords:** Inadequate endometrial tissue, Endometrial sampling, *MedGyn*® pipette, Insufficient endometrial tissue

## Abstract

**Introduction:**

Women presenting with abnormal uterine bleeding needs careful and thorough assessment including ultrasound examination of endometrium and histopathological assessment of the endometrial tissues. The objective of this cross-sectional study was to determine the rate and the factors associated with inadequate endometrial tissues after endometrial sampling using *MedGyn*® pipette among Bhutanese women at the colposcopy clinic, Jigme Dorji Wangchuck National Referral Hospital (JDWNRH), Bhutan.

**Methods:**

This cross-sectional study was conducted at the colposcopy clinic, JDWNRH, Thimphu between October, 2021 and March, 2022. Women included in this study underwent endometrial sampling using *MedGyn*® pipette without anesthesia as an office procedure. Data were collected using an interviewer-administered questionnaire and results extracted into a structured pro forma. The histopathology reports were extracted from the Department of Pathology and Laboratory Medicine, JDWNRH using the unique Bhutanese citizenship identity card number of the study participants.

**Results:**

Inadequate endometrial tissues were noted in 27% (33 out of 122 cases). Among 89 patients with an adequate endometrial tissue, histologic results were normal in 30 (33.7%), benign pathology in 22 (24.7%), atrophy in 10 (8.2%), and hyperplasia in 27 (30.3%). In a univariate analysis, menopausal state (OR 1.6, 95% CI 0.708–3.765), overweight and obese (OR 1.6 95% CI 0.640–3.945), unemployed (OR 1.7, 95% CI 0.674–1.140), nulliparous (OR 1.7, 95% CI 0.183–15.816), primipara (OR 5.1, 95% CI 0.635–40.905) and use of hormonal contraception (OR 2.1, 95% CI 0.449–10.049) were associated with increased risk of inadequate endometrial tissues. On multivariate regression analysis, nulliparity (OR 1.1, 95% CI 0.101–12.061), overweight and obesity (OR 1.4, 95% CI 0.490–3.917), use of hormonal contraceptives (OR 2.2, 95% CI 0.347–13.889), and junior surgeons (OR 1.1, 95%CI 0.463–2.443) were found to be associated with inadequate endometrial tissues. However, the above associations were not statistically significant (*p* > 0.05).

**Conclusion:**

The rate of inadequate endometrial tissue following endometrial sampling using *MedGyn*® pipette was 27.0%. Factors associated with an increased risk of inadequate endometrial tissue after endometrial sampling were menopausal state, overweight and obese, unemployed, nulliparous, primipara and use of hormonal contraception.

**Supplementary Information:**

The online version contains supplementary material available at 10.1186/s12905-024-03047-6.

## Introduction

Abnormal uterine bleeding (AUB) is a common condition affecting reproductive aged women with significant social and economic impact [1]. The FIGO classification of abnormal uterine bleeding (AUB) as PALM-COEIN (*P*olyp; *a*denomyosis; *l*eiomyoma; *m*alignancy and hyperplasia; *c*oagulopathy; *i*atrogenic; and *n*ot yet classified) can be used by clinicians, investigators, and patients to facilitate patient care, communication and research [2].

Careful evaluation with history and clinical examination including speculum and bimanual examination will often direct to the cause of AUB, further assessment and treatment [1]. Endometrial sampling is not required in all patients with AUB. Patients should be selected for endometrial sampling based on the presence of risk factors for endometrial hyperplasia and cancer [2]. SOGC (society of obstetrician and gynaecologist of Canada) recommends transvaginal ultrasonography as first line imaging modality for AUB, if imaging is indicated. The society recommends office endometrial biopsy over dilatation and curettage in women with AUB [1].

Endometrial sampling is a safe and cost-effective office procedure for sampling endometrial tissue in women with AUB. This endometrial tissue would provide a wide range of morphologic patterns; normal and abnormal including endometrial hyperplasia and endometrial cancer [3–5].

Various kinds of endometrial samplers such as *MedGyn*®, *Endocell®, Endocurette*®, *Endosampler*®, *Explora Sampler, Pipelle®*^,^*Tao brush*, and *Li brush* are used in clinical practice to obtain endometrial tissues for histopathological diagnosis [6–11]. Studies have shown high agreement between endometrial sampling and conventional dilatation and curettage to obtain endometrial tissues for histopathological diagnosis [9, 12]. The rate of inadequate endometrial tissues for histopathological diagnosis after endometrial sampling varies between 16% and 80.43% [6–8, 11, 13–16], which must have resulted from the difference in study design, study centre types, diagnostic criteria for inadequate endometrial tissues, and types of service provider [6, 8, 11, 13–16].

In Bhutan, women with AUB or other suspected pathologies of endometrium undergo either endometrial sampling as office procedure in the colposcopy clinic without anaesthesia or dilatation and curettage under some form of anaesthesia in the operation theatre to obtain endometrial tissues for histopathological assessments. The objective of this study was to describe the sociodemographic profiles, histopathology, rate and factors associated with inadequate endometrial tissues among Bhutanese women who had undergone endometrial sampling using *MedGyn*® pipette at the colposcopy clinic, JDWNRH, Bhutan.

## Materials and methods

### Study population

This was a cross sectional study conducted at the colposcopy clinic, JDWNRH between October 2021 and March 2022. Study participants were recruited by convenience sampling method.

All the women who underwent endometrial sampling using *MedGyn*® pipette in the colposcopy clinic were included for the study. Those women who were pregnant, already on treatment for endometrial hyperplasia, who had received pelvic radiation, failed endometrial sampling and those women on tamoxifen therapy were excluded from the study. Informed written consent was obtained from the study participants. Sociodemographic characteristics (age, BMI, education level, occupation, marital status, parity, breastfeeding and smoking history, regularity of menstrual cycle and the use of hormonal contraceptives, indications and medical disorders) were collected using an interviewer administered questionnaire. The histopathology reports were extracted from the Department of Pathology and Laboratory Medicine, JDWNRH using the unique Bhutanese citizenship identity card number of the study participants.

### Sample size

The rate of inadequate endometrial tissues for histopathological diagnosis after endometrial sampling varies between 16% and 80.43% [6–8, 11, 13–16]. The average rate of inadequate endometrial sampling is 48.2%, and this average rate is taken as the rate for the population (p1 = 48.2%). In the present study, the sample size is 122 (*n* = 122); and the rate of inadequate endometrial sampling in the current study is 27% (p2 = 27%). The post hoc power calculation was performed using online software (https://clincalc.com/stats/Power.aspx ) at type I error (α = 0.05). The post hoc power of the study is 99.9%.

### Local setting

The health services in Bhutan are provided through a three-tiered structure: national and regional referral hospital at the tertiary level, general or district hospitals at secondary level, and basic health units (BHU), sub-posts and outreach clinics (ORCs) at the primary level. The regional referral hospitals treat patients referred from the district hospitals under their jurisdiction and further refer cases to the national hospital as needed, serving as a link between the district hospitals and the national referral hospital [17].

JDWNRH is a tertiary care and teaching hospital located in Thimphu, Bhutan. In this hospital, gynaecologists see those women with gynaecological complaints in the gynaecology OPD. Those women with clinical indication to assess endometrial thickness undergo either transvaginal sonography (TVS) or transabdominal sonography (TAS) at the gynaecology OPD or in the Department of Radiology and Imaging Services, JDWNRH. Women with thickened endometrium or with normal endometrial thickness with clinical indications to assess the histopathology of endometrium undergo endometrial sampling as an office procedure without anaesthesia or formal dilatation and curettage under anaesthesia in the operation room.

### Endometrial sampling and histopathology

In this study, all the endometrial sampling were performed as an office procedure by five consultant gynaecologists at the colposcopy clinic, JDWNRH without anaesthesia.

Two senior nurses (DB, MC) in the colposcopy clinic were involved in preparing women for endometrial sampling. They confirmed indications, and contraindications for endometrial sampling, provided pre-procedure briefing and obtained written informed consent for the procedure. Tablet misoprostol 400 micrograms was administered sublingually about 3–4 h prior to endometrial sampling procedure.

Woman was placed in the lithotomy position, bimanual examination performed as a usual procedure to assess uterine size, position and adnexal pathology (but not recorded for the purpose of this study as these details were not included in the current study protocol). Then, sterile Cusco’s bivalve vaginal speculum was inserted, cervix and vaginal fornixes cleaned with betadine solution (10%), anterior lip of cervix was held with single toothed volsellum, and uterine sound gently inserted to ascertain uterine position and the length of utero-cervical canal length.

Once the length and position of uterus was ascertained, endometrial sampling using *MedGyn*® pipette (RB Medical, Alton Road Industrial Estate, Unit 2, Ross-on-Wye, Herefordshire, HR9 5ND, UK, length 23.5 cm, O.D: 3.1 mm) was performed. The pipette was inserted till the fundus of uterine cavity, negative pressure created, and rotated 360 degrees while pushing forth and back. This procedure was repeated till adequate endometrial tissues were obtained. The speculum was removed gently, and women kept under observation for 10–15 min, and then sent home if she had no other complaints.

The endometrial tissues were preserved in 10% formalin and labelled with patient details. Histopathological examination was done in the Department of Pathology and Laboratory Medicine of JDWNRH. Histopathology examination was done in the standard method, and reported. The terminology and diagnostic criteria are based on the WHO Classification of Female Genital Tumors, 5th edition.

### Statistics and data analysis

Data were double entered, validated and analysed using EpiData (Version 3.1 for entry and version 2.2.2.183 for analysis, EpiData Association, Odense, Denmark). Additional analysis was performed using STATA (version 13.1, StataCorp LP USA). The Kolmogorov-Smirnov analysis was performed to assess the normality of continuous variables. Normally distributed continuous variables of sociodemographic and clinical characteristics were compared using t-test. Non-normally distributed two independent variables were compared using the Mann-Whitney test and expressed as medians (IQR). The chi-square or Fisher’s exact test was used to analyze categorical variables.

A univariate and multivariate logistic regression analysis was used to ascertain the factors responsible for inadequate endometrial tissues. A *p* < 0.05 was considered statistically significant with a confidence interval of 95%. The criteria for diagnosing inadequate endometrial tissues was a lack of any tissue fragments containing both glands and stroma.

## Results

### Socio-demographic characteristics of study population

The baseline sociodemographic characteristics of study population are presented in Table 1. Among 122 women who underwent endometrial sampling using *MedGyn*® pipette, inadequate endometrial tissues were present in 27% (33/122). Women in inadequate and adequate groups have a similar demographic characteristic in terms of median age, BMI (body mass index), marital status, occupation, menstrual cycle, occupation, history of smoking and hormonal contraceptive use, parity and history of breast feeding. However, there were more illiterate women in inadequate endometrial sample as compared with adequate samples, which was statistically significant (p 0.047).

**Table 1:** Socio-demographic and clinical characteristics of women who underwent endometrial sampling using *MedGyn*® pipette at JDWNRH ^a^ (*n* = 122)

Features	Total n (%)	Inadequate sample (*n* = 33)	Adequate sample (*n* = 89)	P-value
**Age**				
Median (IQR)	46.5 (42.0-51.2)	48 (42–54)	45 (42–50)	0.205
**BMI** ^**b**^				
Underweight	3 (2.5)	0 (0.0)	3 (3.4)	0.525
Normal	35 (28.7)	8 (24.2)	27 (30.3)	
Overweight	49 (40.2)	16 (48.5)	33 (37.1)	
Obese	35 (29.1)	9 (27.3)	26 (29.2)	
Median (IQR)	27 (23–31)	28 (25–30)	27 (23–31)	0.483
**Marital status**				
Unmarried	1 (0.8)	0 (0.0)	1 (1.1)	0.918
Married	105 (86.1)	29 (87.9)	76 (85.4)	
Divorced	9 (7.1)	2 (5.3)	7 (7.9)	
Widow	7 (5.5)	2 (5.3)	5 (5.6)	
**Educational status** ^**c**^				
Illiterates	56 (45.9)	20 (60.6)	36 (40.4)	0.047
Literate	66 (54.1)	13 (39.4)	53 (59.6)	
**Occupation**				
Housewife	67 (4.9)	20 (60.6)	47 (52.8)	0.584
Civil servant	18 (14.8)	4 (12.1)	14 (15.7)	
Private office	6 (4.9)	0 (0.0)	6 (6.7)	
Business	15 (12.3)	4 (12.1)	11 (12.4)	
Farmer	16 (13.1)	5 (15.2)	11 (12.4)	
**Menstrual cycle**				
Regular	107 (87.7)	31 (93.9)	76 (85.4)	0.202
Irregular	15 (12.3)	2 (6.1)	13 (14.6)	
**Use of hormonal contraceptives** ^**d**^				
Yes	7 (5.7)	3 (9.1)	4 (4.5)	0.332
No	115 (94.3)	30 (90.9)	85 (95.5)	
**Parity**				
Nullipara	5 (4.1)	1 (3.0)	4 (4.5)	0.224
Primipara	13 (10.7)	1 (3.0)	12 (13.5)	
Multipara	104 (85.2)	31 (93.9)	73 (82.0)	
Parity: Median (IQR)	3 (2–4)	3 (2–4)	3 (2–4)	0.15
**Breastfeeding**				
Yes	79 (64.8)	19 (57.6)	60 (67.4)	0.312
No	43 (35.2)	14 (42.2)	29 (32.6)	
**Smoking**				
Yes	8 (5.7)	1 (3.0)	6 (6.7)	0.434
No	115 (94.3)	32 (97.0)	83 (93.3)	
**Medical disorders**				
None	65 (53.3)	15 (45.5)	50 (56.2)	0.257
Obesity ^e^	22 (18.0)	5 (15.2)	17 (19.1)	
Hypertension (HTN)	15 (12.3)	7 (21.2)	8 (9.0)	
HTN & Obesity	6 (4.9)	4 (4.5)	2 (6.1)	
Diabetes and HTN	5 (4.1)	3 (3.4)	2 (6.1)	
DM, HTN and obesity	1 (0.8)	1 (3.0)	0 (0.0)	
Others ^f^	8 (6.6)	1 (3.0)	7 (7.9)	
**Histopathological diagnosis**				< 0.001
Normal	30 (24.6)	0 (0.0)	30 (33.7)	
Benign pathology ^g^	22 (18.0)	0 (0.0)	22 (24.7)	
Atrophy	10 (8.2)	0 (0.0)	10 (8.2)	
Hyperplasia	27 (22.1)	0 (0.0)	27 (30.3)	
Inadequate tissues	33 (100.0)	0 (0.0)	33 (27.0)	

^a^ Jigme Dorji Wangchuck National Referral Hospital; SD, standard deviation; ^b^ Body mass index (Weight in kilograms/Height in meter squared); ^c^ includes primary to master level education and non-formal education; ^d^ oral contraceptive pill. ^e^ Obesity includes women with BMI > 30 kg/m^2^; ^f^ Other medical disorders include hypothyroidism, epilepsy, rheumatic heart disease, and migraine. ^g^ includes disordered, polyp, glomus endometritis

#### Factors associated with inadequate endometrial tissues after endometrial sampling

Adequacy for endometrial tissues was taken as the dependent variable. The sociodemographic and clinical characteristics of the patients were taken as the independent factors affecting the endometrial tissues adequacy. The results of univariate and multivariate logistic regression analysis are shown in Table 2.

In a univariate analysis, menopausal state, overweight and obese, unemployed, nulliparous, primipara and use of hormonal contraceptives were associated with increased risk of insufficient endometrial tissues after endometrial sampling using *MedGyn*® pipette.

In a multivariate regression analysis, nulliparity, overweight and obesity, use of hormonal contraceptives, and junior surgeons were found to have odds ratio greater than one. However, the confidence interval doesn’t state that it is statistically significant (*p* > 0.05).

In contrast, women with irregular menstrual cycles are 50% more likely to have adequate endometrial tissues after endometrial sampling.

**Table 2:** Univariate and multivariate binary logistic regression analysis performed to identify the factors associated with an inadequate endometrial tissue after endometrial sampling using *MedGyn*® pipette at JDWNRH (n=122)

Independent variables	Univariate analysis	p-value	Multivariate analysis	p-value
	OR (95% CI)		OR (95% CI)	
Age	0.9 (0.918 − 0.1006)	0.091	0.9 (0.882–1.033)	0.251
Premenopausal age	1		1	
Menopausal state	1.6 (0.708–3.765)	0.250	0.6 (0.152–2.344)	0.460
Married	1		1	
Single or unmarried	0.8 (0.243–2.676)	0.725	0.7 (0.206–2.977)	0.721
Normal BMI	1		1	
Overweight and obese	1.6 (0.640–3.945)	0.318	1.4 (0.490–3.917)	0.539
Employed	1		1	
Unemployed	1.7 (0.674–1.140)	0.268	1.1 (0.356–3.459)	0.858
Multipara	1		1	
Nullipara	1.7 (0.183–15.816)	0.642	1.1 (0.101–12.061)	0.937
Primipara	5.1 (0.635–40.905)	0.125	-	-
Regular menstrual cycle	1		1	
Irregular cycle	0.4 (0.080–1.770)	0.216	0.5 (0.090–3.417)	0.524
Non-hormonal contraceptive	1		1	
Hormonal contraceptive use	2.1 (0.449–10.049)	0.342	2.2 (0.347–13.889)	0.403
Smoking	0.4 (0.050–3.773)	0.446	0.8 (0.136–5.070)	0.839
Breastfeeding	0.7 (0.289–1.490)	0.314	0.6 (0.252–1.670)	0.37
No medical disorders	1		1	
Obesity	1.0 (0.322–3.228)	0.973	1.1 (0.301–4.067)	0.879
Hypertension	0.3 (0.107–1.1010)	0.072	0.4 (0.125–1.504)	0.188
Hypertension and obesity	0.6 (0.100-3.604)	0.577	0.3 (0.129–2.443)	0.242
DM and hypertension	0.5 (0.069–2.949)	0.405	0.3 (0.039–7.947)	0.560
Senior gynecologists	1		1	
Junior gynecologists	1.0 (0.442–2.193)	0.970	1.1 (0.463–2.443)	0.885

Dependent variables (inadequate sample = 1 vs. adequate sample = 2)

## Discussion

In this cross-sectional study conducted among 122 women who underwent endometrial sampling using *MedGyn*® pipette as office procedure due to various gynaecological indications at the national referral hospital of Bhutan, 27% (33) women had inadequate endometrial tissues for histopathological diagnosis. The rate of inadequate endometrial tissues for histopathological diagnosis after endometrial sampling varies widely in the literature, ranging from 16 to 80.43% [6–8, 11, 13–16]. The difference in the rates of inadequate endometrial tissues may result from difference in study design, diagnostic criteria for inadequate endometrial tissues, and types of service provider. Multi-centre studies have shown lower rates [14, 15] than single centre studies [6, 8, 11, 13, 16].

The rate of inadequate endometrial tissues after endometrial sampling is affected by numerous factors such as age, menopausal status, indications, parity, body mass index, endometrial thickness, and experience of service providers [7, 8, 11]. Indications of endometrial sampling had a significant influence on adequacy of endometrial tissues (*p* = 0.02), with abnormal uterine bleeding yielded highest percentage of diagnostic samples (88.8%) whereas, abnormal endometrial imaging resulted in highest non-diagnostic outcomes (37.25%) [7]. However, patients age and menopausal status were not found to be associated with inadequate endometrial tissues after endometrial sampling in some studies [18].

In a previous study among women with inadequate endometrial tissues, further assessment revealed an endometrial malignancy in 3 out of 66 women (0.05%) and atypical hyperplasia in 1 (0.02%). Authors have recommended for further endometrial sampling in postmenopausal bleeding women with a non-reassuring endometrial thickness [14]. In the present study, 33 women with insufficient endometrial tissues were not further evaluated with repeat endometrial sampling. They are kept under routine surveillance for symptoms and signs of endometrial cancer. If their symptoms persist, concerned gynecologist will follow up the patient with ultrasound assessment of endometrial thickness with either endometrial sampling or conventional dilatation and curettage under anesthesia. This clinical judgement is left up to the individual gynecologist in our setting.

In this study, univariate analysis showed menopausal state, overweight and obese, unemployed, nulliparous, primipara and use of hormonal contraceptives were associated with increased risk of insufficient endometrial tissues. Whereas, multivariate regression analysis showed nulliparity, overweight and obesity, use of hormonal contraceptives, and junior surgeons were associated with increased risk of inadequate endometrial tissues. However, these associations were not statistically significant (*p* > 0.05).

In this study, univariate analysis showed menopause status as a predictor of inadequate endometrial tissues (OR = 1.6, 95% CI = 0.708–3.765), which is in line with previous studies [8, 10, 11, 18]. Multivariate analysis showed that the menopausal status, and endometrial thickness of less than 8 mm were significant independent factors associated with an increased risk of insufficient endometrial tissues after endometrial sampling [11].

The histopathological diagnosis of sufficient endometrial tissues was variable across the studies. In a single centre study conducted by Aue-aungkul A. et al. [11], histologic results in the included 51.9% normal pathological endometrium, 7.3% endometrial polyps, 11.6% endometrial hyperplasia, and 4.7% endometrial cancer. In the present study, histopathological diagnosis was normal in 33.7%, benign pathologies such as disordered, polyp, glomus endometritis in 24.7%, atrophied endometrium in 8.2%, and hyperplasia in 30.3%.

The rate of insufficient endometrial tissues is variable depending on the type of endometrial sampler used. Endometrial sampling performed by using *Endocell* (disposable endometrial sampler tube, CooperSurgical, Inc., Trumbull, CT, USA) which required only a manual suction to extract a sample from the endometrium reported inadequate endometrial tissues rate of 28.8% (95% CI = 23.0–35.0) [11]. In a retrospective review among postmenopausal women in Turkey, the rate of inadequate endometrial tissue using Karman Cannula was 25.9% (143 out of 614 postmenopausal women with either postmenopausal bleeding or incidental finding of increased endometrial thickness in the ultrasound) [13].

In this study, multivariate analysis showed that the endometrial sampling performed by junior gynaecologists (with working experience of less than 15 years) was found to have higher association (without statistical significance) with inadequate endometrial tissues as compared with senor gynaecologist with working experience of more than 15 years (OR = 1.1, 95% CI = 0.463–2.443, *p* = 0.885). A study done in the Medical University of Warsaw, Poland concluded that the effectiveness of endometrial sampling procedure is independent of doctors experience [7].

The findings from this study indicates that endometrial sampling by using *MedGyn*® pipette yields a similar inadequate endometrial tissue as described in the literature, and we should continue using this method of endometrial sampling. This procedure can be done safely in the outpatient setting without anesthesia with subsequent reduction in cost and improved patients convenience.

This is the first study on inadequate endometrial tissues after endometrial sampling in the Bhutanese context which provides a baseline data, and way forwards for plans and strategies for improvement. The major limitation of the present study was inability to gather data about the endometrial thickness, because many women reported either without undergoing ultrasound examination or there was no mention of endometrial thickness in those women who had ultrasound assessment. In addition, some women had transvaginal ultrasound and some had transabdominal ultrasound performed by different ultrasound technicians or gynecologist whose observation were subjective. With the insights gained from this study, we would like to make some recommendations to reduce the rate of insufficient endometrial tissues after endometrial sampling in our context: proper selection of patients with genuine clinical indications supported by transvaginal ultrasound assessment of endometrial thickness prior to advising endometrial sampling. Since the rate of insufficient endometrial tissues are variable depending on individual gynecologist, we recommend to have a standard guidelines in place for all the gynecologists to follow and to teach the residents. If possible, authorities should ensure availability of ultrasound machine with transvaginal probe in every gynecologist’s chamber so that the gynecologist can assess endometrial thickness.

## Conclusion

From the present study, we conclude that the rate of inadequate endometrial tissues after endometrial sampling with *MedGyn*® pipette as an office procedure is comparable with the similar studies done elsewhere in other countries. However, there was no identified factors with statistically significant associated with inadequate endometrial tissues in our setting. As the study sample size is small, further study with bigger sample size and measurement of endometrial thickness would be valuable to come to a definite conclusion of risk factors associated with inadequate endometrial tissues after endometrial sampling in the context of Bhutan.

### Electronic supplementary material

Below is the link to the electronic supplementary material.

Supplementary Material 1

## Data Availability

The datasets generated and/or analyzed during the current study are not publicly available due personal information protection, patient privacy regulation, and medical institutional data regulatory policies, etc., but are available from the corresponding author on reasonable request and with permission of the Research Ethics Board of Health, Ministry of Health, Thimphu, Bhutan.
